# Tuning Relative Polypeptide Expression to Optimize Assembly, Yield and Downstream Processing of Bispecific Antibodies

**DOI:** 10.3390/antib7030029

**Published:** 2018-08-10

**Authors:** Giovanni Magistrelli, Guillemette Pontini, Yves Poitevin, Pauline Malinge, Jérémie Bourguignon, Florence Gauye, Elise Fleury, Nicolas Plèche, Lydia Galissaires, Nicolas Fischer

**Affiliations:** Novimmune SA, 14 Chemin des Aulx, 1228 Plan-les-Ouates, Switzerland; gmagistrelli@novimmune.com (G.M.); gpontini@novimmune.com (G.P.); ypoitevin@novimmune.com (Y.P.); pmalinge@novimmune.com (P.M.); jbourguignon@novimmune.com (J.B.); fgauye@novimmune.com (F.G.); efleury@novimmune.com (E.F.); npleche@novimmune.com (N.P.); lgalissaires@novimmune.com (L.G.)

**Keywords:** affinity chromatography, bispecific antibody, codon optimization, co-expression, transient transfection, stable pools, product related impurities

## Abstract

Bispecific antibodies (bsAbs) are often composed of several polypeptide chains that have to be expressed adequately to enable optimal assembly and yield of the bsAb. κλ bodies are a bispecific format with a native IgG structure, composed of two different light chains that pair with a common heavy chain. Introduction of non-optimal codons into the sequence of a particular polypeptide is an effective strategy for down modulating its expression. Here we applied this strategy but restricted the modification of the codon content to the constant domain of one light chain. This approach facilitates parallel optimization of several bsAbs by using the same modified constant domains. Partial sequence de-optimization reduced expression of the targeted polypeptide. Stable cell pools could be isolated displaying increased bispecific antibody titers as well as changes in the abundance of undesired by-products that require elimination during downstream processing. Thus, modulating the relative expression of polypeptides can have a significant impact on bsAb titer and product related impurities; which are important factors for large scale manufacturing for clinical supply.

## 1. Introduction

In the last two decades, bispecific antibodies (bsAbs) have emerged as a new class of therapeutic molecules. The key difference to a standard antibody is their capacity to simultaneously engage two antigens [1]. This property enables unique modes of action such as directing cells of the immune system to cancer cells or improving trafficking across the blood brain barrier [2,3]. The recent approval of a third bsAb for the treatment of haemophilia A demonstrates that, beyond oncology, the potential of bsAbs can be applied to different therapeutic areas [4]. Protein engineering efforts have led to a stunning variety of different possible approaches for the generation of bsAbs, with a current estimate of more than 100 different formats [5,6]. These can be classified in different ways such as size, valency of binding sites, overall molecular structure and similarity to a standard IgG format. Another important difference between these formats is the number of chains that are co-expressed to assemble the bsAb which can vary between 1 to 4 polypeptides [7]. For example, Blincyto, the approved anti-CD3, anti-CD19 (CD3xCD19) bispecific T cell engager (BiTE) is a tandem single chain variable fragment scFv format composed of a single polypeptide. Another example is Hemlibra, which is the recently approved bsAb with Factor VIII mimetic activity that has a standard IgG structure and requires the co-expression of three polypeptides: two heavy chains and a single common light chain capable of pairing with both heavy chains [8]. Another example requiring the expression of four different chains is the CrossMab technology, incorporating interface alterations and domain swapping leads to that preferential assembly of the bispecific antibody [6]. When a bsAb format requires co-expression of three or four polypeptides, by-products resulting from incorrect chain pairing are produced, regardless of the format and engineering approach. Although these by-products may be considered as minor contaminants, it can be challenging to remove them during downstream processing [5,9]. 

In this context, differences in the expression level of each chain composing the bsAb can lead to significant increase in by-product levels, reducing bsAb yield and making downstream purification even more challenging. Obtaining cell lines with appropriate expression of each chain of the bsAb is particularly important for large scale manufacturing in order to maximize yield and purity of the desired bispecific molecule. Although the stoichiometric expression of each polypeptide could *a priori* be considered as the optimal situation for assembly, this is not always the case. Indeed, even in the simpler context of standard antibody expression, the level of free light chains in the culture supernatant correlates with antibody titer, indicating that an excess of light chain is required to achieve high IgG secretion [10,11]. 

An important characteristic of the κλ body format is its native human IgG structure as it does not incorporate any mutations or foreign sequences [12]. A κλ body is composed of two different light chains, one kappa and one lambda, that pair with two copies of the same heavy chain. As a result, in addition to assembly of bispecific κλ bodies (IgG κλ), two monospecific by-products incorporating either two kappa chains (IgGκκ) or two lambda chains (IgGλλ) are also generated upon co-expression of the common heavy chain and the two light chains. Monospecific and bispecific antibody formation is dependent on the random assembly of heavy and light chains. It is theoretically anticipated that the equivalent expression and random association of a common heavy chain, a kappa light chain and a lambda light chain leads to the secretion of three antibody forms, IgG κλ, IgGκκ and IgGλλ, following a 2:1:1 ratio. Indeed, based on the expression and purification of over 100 different κλ bodies, we found that the bsAb represents 40 to 50% of secreted IgG when the expression of each light chain falls within a range of 30 to 70% of total light chain content. Thus it is only in rare cases that unbalanced expression of the two light chains is such that the percentage of bsAb is significantly affected. However, beyond discovery and bsAb characterization, when considering large scale expression and manufacturing, maximizing yield via fine tuning the expression of different chains might become particularly important. We previously demonstrated that altering the expression of one light chain could significantly shift the distribution between bsAb and by-products [13]. Interestingly, reducing the expression of the most abundant light chain was significantly more effective than optimizing the codon usage to increase expression of the underrepresented light chain. This was achieved by introducing codons having a lower frequency in mammalian cells into the DNA sequence, encoding the variable and constant regions of the over expressed light chain. This strategy allowed a more balanced expression of the light chains resulting in significant improvement in yield for a suboptimal producing candidate. 

Here we further applied codon de-optimization to other candidates already considered as suitable in terms of product yield. Fine tuning the expression of the polypeptides both maximized the yield and minimized by-product contamination facilitating downstream processing. Importantly, we demonstrate that modifying the expression by restricting sequence alterations to the constant region of the light chains will simplify optimization of multiple candidates in parallel.

## 2. Materials and Methods

### 2.1. Codon Optimization and De-Optimization

Non-optimal codon sequences for mammalian cells were generated by GeneArt^®^ (GeneOptimizer^®^ software, Regensburg, Germany) on the gene encoding of the lambda constant light chain. Inhibitory motifs (such as possible splice sites) have been removed in all sequences. 

### 2.2. Plasmid Generation

The common heavy chain, the kappa and the lambda light chains were cloned into a single pNOVI expression vector containing three expression cassettes under the transcriptional control of the human cytomegalovirus promoter. 

First, second and third promoter drove, respectively, the expression of the K2 kappa light chain which binds to human cluster of differentiation 47 (hCD47), the common heavy chain, and the different lambda light chains (O30, O35 and O41) which target human mesothelin (hMSLN). Different variants were obtained by cloning these variable lambda domains upstream of either wild type or de-optimized constant lambda domains (Figure S1).

### 2.3. Transient IgG Expression in Mammalian Cells

Transformed Human Embryo Kidney monolayer epithelial cells (PEAK cells; Edge Bio, La Jolla, CA, USA) were maintained in 5% CO_2_ at 37 °C in a humidified atmosphere in DMEM (Invitrogen, Carlsbad, CA, USA) containing 10% FCS (Sigma-Aldrich, St Louis, MO, USA) and supplemented with 2 mM glutamine (Sigma-Aldrich, St Louis, MO, USA), referring to complete DMEM. One day before transfection, confluent cells were splited 1:4 in T175 flasks (Nunc™ Cell Culture Treated Flasks with Filter Caps, Thermo Scientific, Waltham, MA, USA) in order to have cells in the exponential growth phase. Transient transfections were performed using a mix containing 30 µg of DNA and 42 µL of Lipofectamine 2000 transfection reagent (Invitrogen, Carlsbad, CA, USA) in 1.4 mL of DMEM for 10^7^ cells per T175 flask in 50 mL of complete DMEM. 

IgG expression was measured using the Octet RED96 instrument with protein A-coated biosensors (Pall ForteBio, Menlo Park, CA, USA). According to antibody concentration, supernatants were harvested 7 to 10 days after transfection.

### 2.4. Stable Pool Generation in CHO-S Cells

CHO-S (Thermo Fisher Scientific, Waltham, MA, USA) cells were routinely cultured in suspension at 2 × 10^5^ cells/mL in CD CHO medium (Sigma-Aldrich, St Louis, MO, USA) and supplemented with 6 mM l-glutamine (Sigma-Aldrich, St Louis, MO, USA) in Erlenmeyer flasks. Cell culture was performed at 37 °C with 5% CO_2_ and 85% relative humidity at 140 rpm. For stable transfection, CHO-S cells in the exponential growth phase were resuspended at 1.43 × 10^7^ cells/mL in CD CHO medium without glutamine and mixed with 40 mg of linearized DNA (Sigma-Aldrich, St Louis, MO, USA) in an electroporation cuvette of 0.4 cm (Bio-Rad, Hercules, CA, USA). After electroporation by single pulse of 300 V, 900 mF and infinite resistance using Gene Pulser XcellTM Electroporation Systems (Bio-Rad, Hercules, CA, USA), cells were immediately added in 50 mL of CD CHO medium without glutamine and distributed in two 6-well plates at 4 mL/well or in two T-75 flasks (Nunc™ Cell Culture Treated Flasks with Filter Caps, ThermoScientific, Waltham, MA, USA)and placed in humidified 10% CO_2_ incubator (Thermo Scientific, Waltham, MA, USA) set at 37 °C. The following day, l-methionine sulfoximine (MSX) (Sigma-Aldrich, St Louis, MO, USA) was added to the culture at 50 mM final concentration for transfected cell selection. After 4 to 5 weeks of growth under selection pressure, pools were assessed for their IgG productivity, and transferred to Erlenmeyer flasks and amplified in suspension. 

### 2.5. Productivity Evaluation by Fed-Batch Overgrowth Cells (FOG)

The productivity of CHO pools was assessed by FOG evaluation. Cells were inoculated at 3 × 10^5^ cells/mL in 50 mL of CD CHO medium supplemented with 300 mg/L of l-cysteine (Sigma-Aldrich, St Louis, MO, USA), 120 mg/L of l-tyrosine (Sigma-Aldrich, St Louis, MO, USA), 50 µM MSX and fed with 15 mL CHO CD EfficientFeed™ B Liquid Nutrient Supplement (Invitrogen, Carlsbad, CA, USA) at day 1. The glucose level was monitored using the GlucCell Glucose Monitoring System (CESCO Bioproduct, Atlanta, GA, USA), and adjusted when necessary. Cells were harvested at day 15 post inoculation or when viability had dropped below 75%. IgG quantitation was measured by Octet.

### 2.6. IgG Purification

After 7–10 and 15 days of antibody production respectively for PEAK cells and CHO cells, the supernatant was harvested, clarified by centrifugation 10 min at 2000 rpm and filtered on a 0.22 µm membrane (Millipore, Burlington, MA, USA). Total IgGs were purified by one affinity chromatography step using the FcXL resin (Life Technologies, Carlsbad, CA, USA) or the MabSelect SuRe resin (GE Healthcare, Chicago, IL, USA) for PEAK and CHO supernatant respectively. A serum-containing medium is used for production in PEAK cells; bovine IgGs do not bind to the FcXL resin, while they bind to the MabSelect SuRe resin. Then, two additional affinity chromatography steps were required to isolate the κλ body and eliminate the two monospecific mAbs: one purification on the KappaSelect resin (GE Healthcare, Chicago, IL, USA) to eliminate the lambda mAbs and one purification on the LambdaFabSelect resin (GE Healthcare, Chicago, IL, USA) to get rid of kappa mAbs. 

An appropriate amount of MabSelect SuRe or FcXL resin was washed three times with (phosphate-buffered saline) PBS (Sigma-Aldrich, St Louis, MO, USA) and resuspended in PBS. The resin was added to the supernatant and the mix was incubated overnight at 4 °C and 15 rpm. Samples were centrifuged at 2200 rpm for 10 min to collect the resin and the flow through was discarded. The resin was washed with PBS and transferred on SigmaPrep^TM^ spin column (Sigma-Aldrich, St Louis, MO, USA). Elution was performed with glycine (Sigma-Aldrich, St Louis, MO, USA) 50 mM at pH 3.0 (Sigma-Aldrich, St Louis, MO, USA). Following purification, the total IgG and the κλ body were formulated into 25 mM histidine (Sigma-Aldrich, St Louis, MO, USA), 125 mM NaCl (Sigma-Aldrich, St Louis, MO, USA) at pH 6.0, by desalting on Amicon Ultra-4 centrifugal filters with membrane Ultracel 50 kDa (Merck Millipore, Burlington, MA, USA) previously equilibrated with formulation buffer (25 mM histidine, 125 mM NaCl at pH 6.0).

The final antibody concentration was evaluated by Nanodrop^®^ (Thermo Scientific, Waltham, MA, USA) 

### 2.7. Characterization of Purified Antibodies

Monospecific antibodies and κλ body distribution and integrity were assessed by electrophoresis, isoelectric focusing gel analysis (IEF), hydrophobic interaction high performance liquid chromatography (HIC-HPLC) and size exclusion high performance liquid chromatography SEC-HPLC. Purified IgGs were analyzed by electrophoresis in denaturing and reducing conditions. The Agilent 2100 Bioanalyzer was used with the Protein 80 kit as described by the manufacturer (Agilent Technologies, Santa Clara, CA, USA). The distribution of the different formats of IgG (monospecific lambda, kappa and bispecific antibody) was determined by isoelectric focusing (Cambrex pH 7–11 IsoGel agarose plates) and HIC-HPLC analysis using ProPac HIC-10 column (Dionex, Sunnyvale, CA, USA). A gradient of mobile phase A (0.01 M sodium phosphate dibasic buffer (Sigma-Aldrich, St Louis, MO, USA) + 1.5 M ammonium sulphate (Sigma-Aldrich, St Louis, MO, USA), pH 3.5) from 100 to 10% and a growing gradient of mobile phase B (0.01 M sodium phosphate dibasic buffer + 10% acetonitrile (Merck KGaA, Darmstadt, Germany), pH 3.5) from 0 to 90% were applied. A blank was performed with mobile phase A, pH 7.0. Aggregate and fragment levels were determined by SEC-HPLC with a Biosep-SEC-s3000 column (Phenomenex, Torrance, CA, USA) using a 200 mM sodium phosphate dibasic buffer, pH 7.0 mobile phase. 

## 3. Results

**Figure 1:**
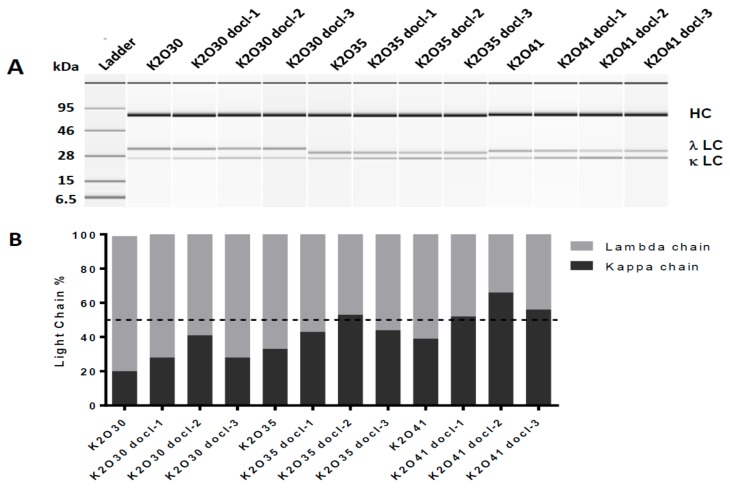

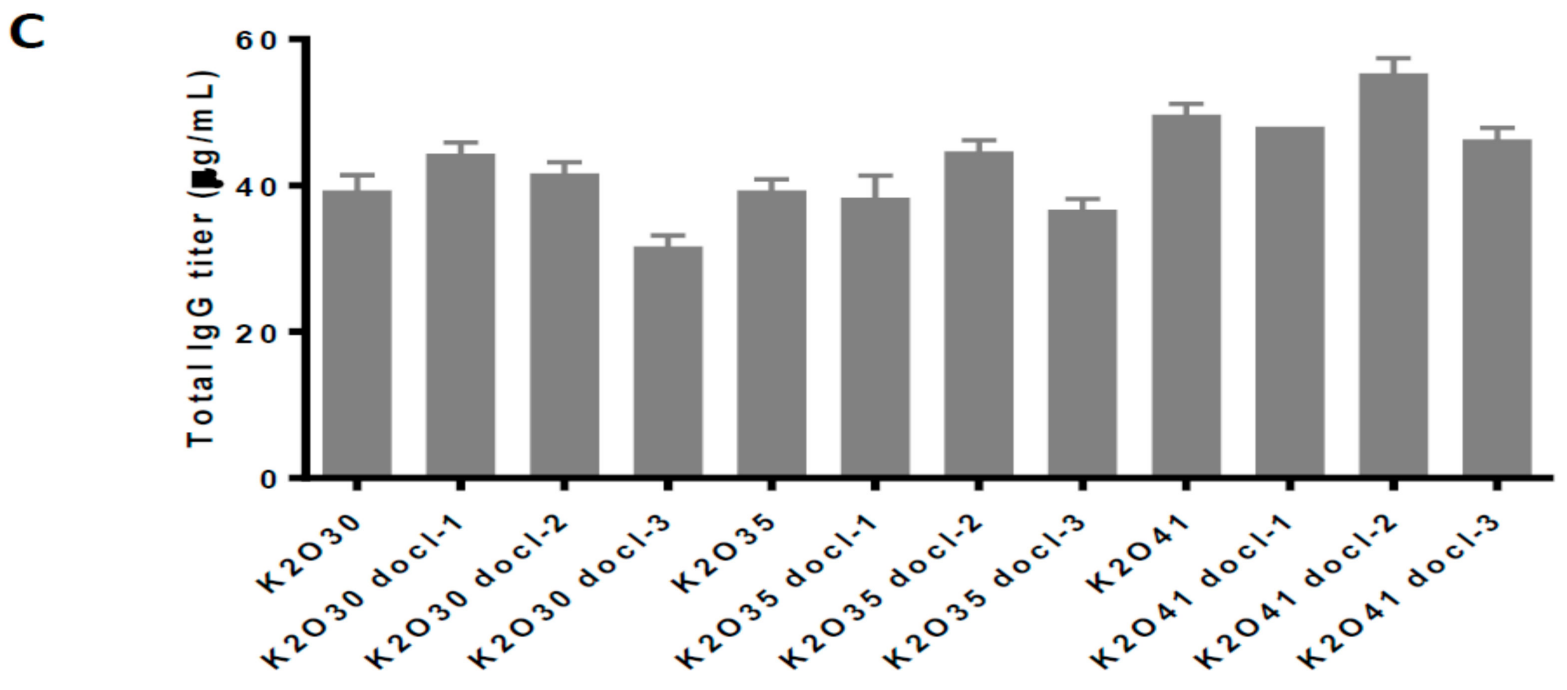
IgG expression of K2O30, K2O35, K2O41 and their respective codon de-optimized variants after transfection in Transformed Human Embryo Kidney monolayer epithelial cells (PEAK cells). (**A**) Electrophoresis using the Agilent Bioanalyzer 2100 for total IgG obtained after the first affinity chromatography step capturing all three IgG forms. The bands corresponding to the common heavy chain (HC), the lambda light chain (λ LC) and the kappa light chain (κ LC) in reducing and denaturing conditions are indicated. (**B**) Ratio of the light chains as determined from the band intensity of the Agilent result. (**C**) Total IgG concentration in the culture supernatant.

**Figure 2:**
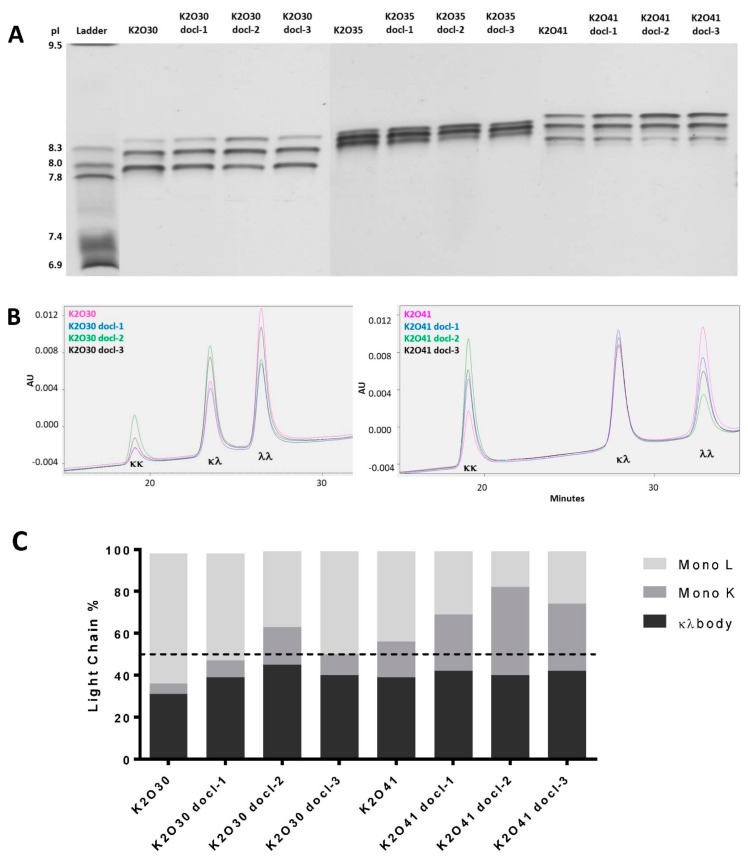
IgG expression after transfection in PEAK cells of K2O30, K2O35, K2O41 and their respective codon de-optimized variants. (**A**) Isoelectric focusing analysis of the purified total IgG samples; the top and bottom bands represent the IgGκκ and the IgGλλ, respectively, the intermediate band corresponds to the IgGκλ (κλ body). (**B**) Analysis by hydrophobic interaction high performance liquid chromatography (HIC-HPLC) of purified total IgG for K2O30, K2O41 and their respective codon de-optimized variants. Peaks corresponding to each of the three forms are indicated. (**C**) Distribution of the three IgG forms analyzed by HIC-HPLC. pI: Isoelectric Point. AU: area under the curve

We have generated 3 κλ bodies (K2O30, K2O35 and K2O41) targeting human CD47 and human mesothelin (MSLN). All three bsAbs incorporate the same anti-CD47 arm (K2) containing a kappa light chain but different anti-MSLN arms (O30, O35 and O41) containing different lambda light chains based on different variable germline genes (Table 1). The common heavy chain and respective light chains of these candidates were transiently co-expressed in mammalian cells using a single plasmid with three independent promoters. After purification of all IgG forms (IgGκκ, IgGκλ, IgGλλ) from the culture supernatant via affinity chromatography, the polypeptides were separated on an Agilent Bioanalyzer. This analysis revealed significantly different light chain contents for each candidate, with 20%, 30% and 38% of kappa light chain incorporated into the different IgG forms of K2O30, K2O35 and K2O41, respectively (Figure 1). Although for these candidates the lambda light chain was more abundant, this is not a general phenomenon as higher kappa light chain content has also been observed for different κλ bodies [14]. Isoelectric focusing gel analysis confirmed the over-representation of the lambda chain for K2O30, K2O35 with high intensity bands corresponding to the monospecific IgGλλ. In contrast, for K2O41 the bands corresponding to IgGλλ and IgGκκ were of comparable intensity, while the bispecific IgGκλ was the most intense, as expected in a situation of more balanced light chain expression (Figure 2A). The IgG form distribution was confirmed and quantified by hydrophobic interaction chromatography (HIC) for K2O30 and K2O41 (Figure 2B; Table 1) but not for K2O35 as the method did not allow for sufficient separation of the three forms. 

### 3.1. Modulation of Light Chain Expression Using Modified Constant Regions

We selected these three κλ bodies as they displayed different degrees of lambda chain overexpression and we aimed at restoring a more balanced expression via a codon de-optimization approach [13]. However, to avoid designing several new sequences for each candidate as previously described, we evaluated the possibility to achieve down modulation while restricting the sequence modification to the constant region of the lambda chain. In this way, the same de-optimized constant regions could potentially be used for several candidates in parallel, thus streamlining the optimization process. We designed three DNA sequences (docl-1, -2 and -3) encoding the constant lambda 2 domain (Figure S1) [15]. Nucleotide changes did not alter the amino acid sequence but introduced codons less frequently used in Chinese hamster ovary (CHO) cells. For each κλ body, three plasmids were generated in which their respective variable lambda sequence was combined with the three modified lambda constant regions. These 12 constructs were transiently transfected into PEAK cells and the total IgG expression levels were overall unaffected (Figure 1C). The different IgG forms were purified by affinity chromatography and analysed for kappa and lambda light chain content as well as IgG form distribution by IEF and HIC (Figure 1 and Figure 2; Table 1). In all cases, the combination of a de-optimized constant region with variable region led to a reduction of the lambda light chain content and concomitant abundance of IgGλλ in the cell culture supernatant. In addition, the impact of a given modified constant domain was similar for the three bsAbs, the docl-2 sequence having a more significant effect on expression, while docl-1 and -3 being relatively equivalent. These results indicate that down regulation of expression can be achieved by altering the codon content of a portion of a polypeptide. In addition, the effects appeared to be consistent for lambda light chains utilizing different germline genes. 

### 3.2. Impact of De-Optimized Constant Regions on Expression in Stable CHO Pools

**Figure 3:**
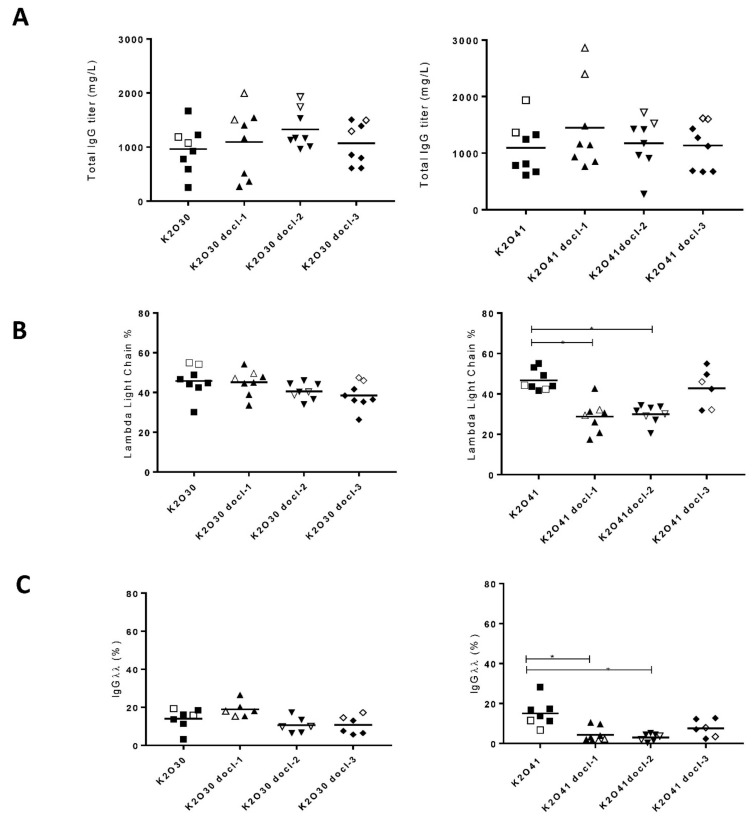

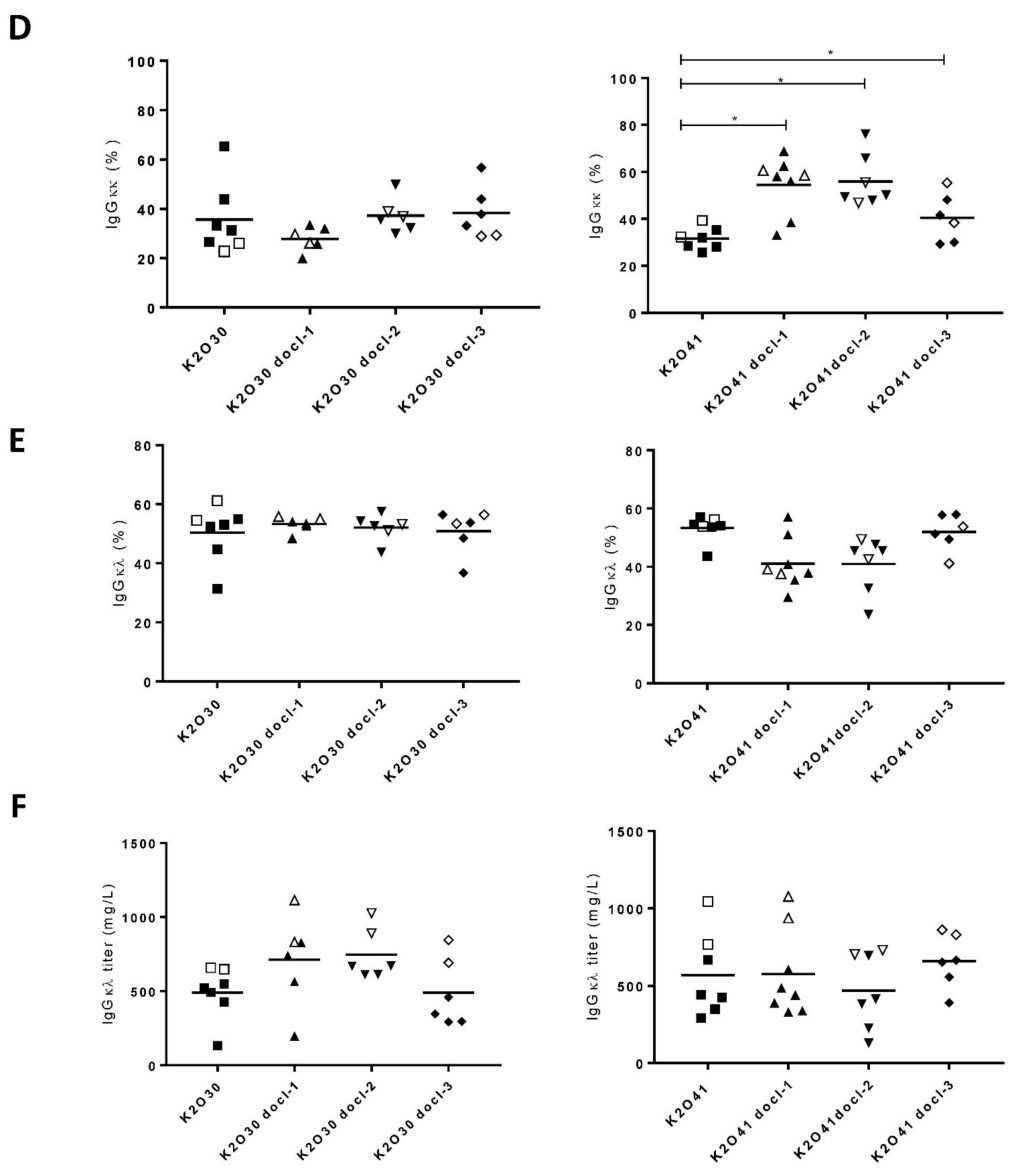
Analysis of IgG ratios and yields in the supernatant of stable CHO pools transfected with K2O30, K2O41 and their respective codon de-optimized variants. Each symbol represents an independent pool. Open symbols indicate the two highest IgGκλ producing pools for each variant. (**A**) Total IgG titers in the supernatants. (**B**) Percentage of lambda light chain determined using the Agilent Bioanalyzer 2100. (**C**–**E**) Percentage of different IgG forms in total IgG after one step of purification analyzed by HIC-HPLC. (**F**) IgGκλ titers in the supernatants. (*) represent a *p* value of < 0.5 using one way analysis of variance.

Expression in stable CHO cell lines is a more relevant system when considering the production of therapeutic proteins such as antibodies. In order to extend the results obtained with transient transfections using PEAK cells, we generated stable CHO pools for K2O30 and K2O41. The original as well as the three de-optimized constructs for each κλ body were introduced into CHO cells by electroporation. Between six and eight stable pools were selected and expanded for each construct. Productivity as well as IgG form distribution were evaluated under fed batch fermentation conditions. The average total IgG titers were similar for all constructs with expected variations often observed between stable pools (Figure 3A). Total IgG were purified by Protein A affinity chromatography and the relative abundance of the kappa and lambda light chains as well as the percentage of different IgG forms were determined. The analysis of light chain content revealed a balanced representation of kappa and lambda light chains in the supernatant of pools harboring the original K2O30 and K2O41 constructs (Figure 3B). This was surprising for K2O30, that under transient transfection conditions led to 80% of lambda light chain assembly (Figure 1B). Nevertheless, the codon de-optimized constant domains also led to a reduction of the lambda light chain content, albeit more markedly for K2O41 (Figure 3B). Another difference compared with transient expression was that the impact of the modified constant domains was different, the docl-1 and docl-3 sequences having more effect in the context of K2O41 and K2O30, respectively. The HIC analysis confirmed the lesser impact of de-optimization on K2O30 compared to K2O41 for which IgGλλ could be reduced to less than 10% with a concomitant increase of IgGκκ more than 50% (Figure 3C,D). The equivalent incorporation of both light chains in the unmodified constructs led to an optimal bispecific assembly and content close, or even above, the theoretical value of 50% (Figure 3E). Indeed, for both K2O30 and K2O41, individual pools reached values of 63% and 57% of IgGκλ content, respectively. As expected, the proportion of K2O30 bispecific remained largely unaffected by de-optimization, reflecting the limited impact on lambda light chain expression. For K2O41, the bispecific content was reduced as the de-optimization led to an excess of IgGκκ secretion creating a more unbalanced situation (Figure 3D). Despite the limited number of pools studied, we were able to identify pools producing over 1 g/L of bispecific IgGκλ under shake flasks conditions for both K2O30 and K2O41 (Figure 3F). 

The bispecific IgGκλ were purified via two additional purification steps using affinity chromatography media binding specifically to the kappa and lambda chains, as previously described [12]. The aggregate levels were consistently low (0.8–2.3%) and not altered by the de-optimization process (Figure S2). The antigen-binding capacity of each arm was also determined using an Octet system and was found to be identical between the original and de-optimized versions [16]. 

### 3.3. Selecting Pools with Favourable Parameters for Large Scale Expression and Downstream Processing

**Figure 4:**
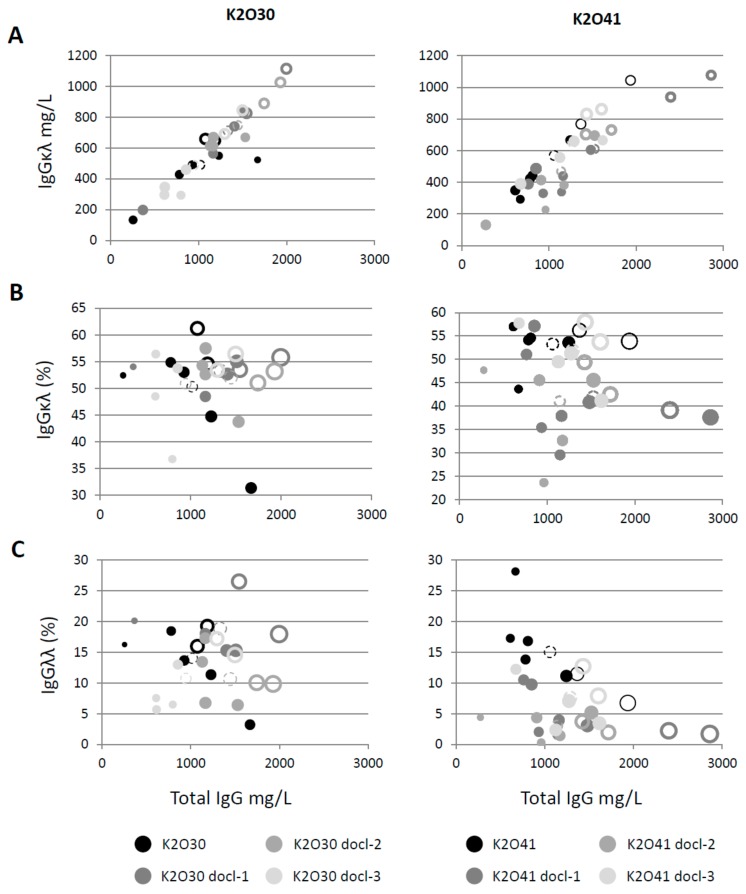
Representation of different parameters analyzed in the supernatants of stable CHO pools K2O30, K2O41 and their respective codon de-optimized variants. (**A**) Correlation between total IgG and IgGκλ titers. (**B**) Representation of total IgG titers, abundance of IgGκλ and IgGκλ titers. (**C**) Representation of total IgG titers, abundance of IgGλλ and IgGκλ titers. In B and C the size of the circles corresponds to the IgGκλ titer. Open circles correspond to the pools represented by open symbols in Figure 3. Circles in dotted lines correspond to the average of all pools for a given construct.

The ultimate objective of large scale expression of a bispecific antibody is to maximize yield of final purified material, while maintaining contaminant and in particular by-products to a minimum. The final yield is affected by total IgG titer, level of bispecific assembly versus by-product assembly as well as efficiency of the downstream process. In the case of the κλ body format, the bispecific titer in the culture supernatant is dependent on total IgG titer and on the % of IgGκλ. However, the first parameter appears to be the most important as shown by the clear correlation between total IgG and IgGκλ titers (Figure 4A). Interestingly, pools showing the highest percentage of IgGκλ did not necessarily show the highest IgGκλ titer (Figure 4B). For instance, amongst the three K2O41 pools expressing close or above 1 g/L of IgGκλ, one pool (K2O41 wt-1) harbouring an unmodified construct secreted 53% of IgGκλ and a total IgG titer of 1.9 g/L (Table S1). In contrast, two pools (K2O41 docl-1-3 and K2O41 docl-1-6) generated with the de-optimized constant lambda domain docl-2 secreted only 35% and 37% of IgGκλ but this suboptimal situation was largely compensated by high total IgG titers of 2.4 and 2.8 g/L, respectively. Another interesting feature of these two pools was the presence of very low levels of IgGλλ (around 2%) (Figure 4C and Table S1). During the downstream processing of κλ bodies, monospecific IgGλλ and IgGκκ were effectively eliminated as they were not retained on affinity columns binding kappa or lambda light chains, respectively. Thus pools in which one by-product (here IgGλλ) is present in a very low amount, enable more efficient use of column capacity and importantly opens up the possibility of removing one affinity purification step from the process. We tested this hypothesis by applying a second affinity purification step to the mixture of IgG forms isolated from the supernatant of K2O41 wt-6 and K2O41 docl-3-5 that contained around 12% of IgGλλ, as well as K2O41 docl-1-3 and K2O41 docl-1-6 that contained only 2% of IgGλλ (Table S1). As anticipated when using LambdaFabSelect resin that binds to the constant region of the lambda light chain, the IgGκκ was eliminated effectively in the flow through (Table S2). The purity of the bispecific IgGκλ was 94% for K2O41 docl-1-3 and K2O41 docl-1-6 having a low initial IgGλλ content, whereas it was of 73% and 86% for K2O41 wt-6 and K2O41 docl-3-5. Elimination of the remaining 13–27% of the monospecific contaminant from the latter samples requires a third affinity step following the generic downstream process established for κλ bodies [12]. In contrast, monospecific contaminants in the 5% range can be eliminated using standard chromatography media or by applying differential elution steps as the IgGλλ containing two lambda constant binding sites is better retained on the affinity column, a strategy that has been described for other bsAb formats [17].

## 4. Discussion

Over the last decade, many options have become available to generate bsAbs with very different formats. In most cases, the objective is to achieve the production of the bsAb from a single cell while minimizing the generation of by-products such as miss-paired chains or partially assembled molecules. In order to achieve this goal, elegant protein engineering approaches have been developed to remodel protein-protein interfaces and promote correct chain pairing [6,7]. When the κλ body format was developed, the main objective was to avoid the introduction of any protein sequence modification to preserve the properties of monoclonal antibodies, such as stability, prolonged circulating half-live and low immunogenicity, that contributed to their success as therapeutic agents. Our approach relies on effective affinity purification of the IgGκλ, that is the only molecule binding to all affinity media used in the downstream purification process, ensuring both high purity and integrity of the purified material. As for other formats that rely on the co-expression of multiple chains, κλ body yield as well as abundance of by-products is affected by the expression and assembly rates of the different polypeptides. Thus, it is important to aim at controlling their relative expression levels, which can be achieved by altering gene copy number, promoter strength or by DNA sequence optimization [13,18,19]. We previously described that the introduction of lower frequency codons into the sequence encoding both the variable and constant domains of a highly expressed light chain could significantly improve bsAb yield. Here we demonstrated that limiting the modification of codon content to a part of the sequence (i.e., the constant domain of the light chain) was also effective in tuning down expression. The first benefit of this modular approach is that it facilitates the evaluation of multiple de-optimized domains in parallel on several bsAb candidates. Our data also indicates that the effect of de-optimized sequences can be different depending on the candidate, reinforcing the need to test multiple combinations. Modular de-optimization is in principle applicable to other bsAb formats or even more widely to the expression of other protein complexes. Furthermore, it has been shown that the removal of lower frequency codons can significantly alter co-translational folding of some proteins by removing pause sites during translation [19,20]. Although we saw no difference in aggregate levels between the different bsAb variants, we cannot exclude that newly introduced low frequency codons might have an effect on translation rate and folding. Thus, another benefit of restricting codon alteration to a single domain is to limit such risks.

We have shown in our previous study that the effects on light chain content and IgG form distribution obtained with different de-optimized variants were consistent between transient transfections using PEAK cells and expression from stable CHO pools [13]. In this study, the results obtained with both systems were also similar for K2O41 but appeared to be different for K2O30. This finding suggests a candidate-dependent effect but might also reflect differences between the cell types used for expression. To improve consistency in future studies, CHO cells can be used for both transient expression and stable cell line generation. Yield, integrity and purity are critical parameters when expressing and purifying therapeutic proteins. BsAbs are complex molecules and their large scale production has been, and remains, far more challenging than standard monoclonal antibodies. In particular, removal of product-related impurities (or by-products) can be difficult [9,17]. We have shown that tuning the expression of one polypeptide enabled the identification of stable transfected CHO cells not only secreting bsAb titers of more than 1 g/L in shake flasks, but also having an unbalanced content of monospecific by-products that can simplify their elimination during downstream processing. Thus, although bsAb titer remains the most important parameter for cell line development, modulating polypeptide expression can also improve other parameters that can have a significant impact when considering large scale manufacturing operations.

## Figures and Tables

**Table 1:** Light chain and lgG form distributions in total lgG after transfection in PEAK cells.

bsAb	V Lambda Germline ^a^	% Kappa	% Lambda	% IgGκκ	% IgGκλ	% IgGλλ
**K2O30 wt**	IGLV1-44	20	80	5	32	63
**K2O30 docl-1**	IGLV1-44	28	72	9	40	51
**K2O30 docl-2**	IGLV1-44	41	59	19	45	36
**K2O30 docl-3**	IGLV1-44	29	71	10	40	50
**K2O35 wt**	IGLV5-45	35	65	NA	NA	NA
**K2O35 docl-1**	IGLV5-45	42	58	NA	NA	NA
**K2O35 docl-2**	IGLV5-45	52	48	NA	NA	NA
**K2O35 docl-3**	IGLV5-45	43	57	NA	NA	NA
**K2O41 wt**	IGLV3-21	38	62	18	39	43
**K2O41 docl-1**	IGLV3-21	51	49	27	43	30
**K2O41 docl-2**	IGLV3-21	63	37	43	40	17
**K2O41 docl-3**	IGLV3-21	55	45	32	43	25

^a^ The germline origin of the gene encoding the variable lambda domain is indicated using the IMGT nomenclature [15]. BsAb: bispecific antibody; IGLV: Immunoglobulin Lambda light chain variable gene; NA: Not applicable; wt: wild type.

## Supplementary Materials

The following are available online at http://www.mdpi.com/2073-4468/7/3/29/s1, Figure S1: (A) Nucleotide sequence alignment for the wild type human constant lambda 2 domain and codon de-optimized variants. All nucleotide sequences encode the same amino acid sequence. (B) Schematic representation of pNOVI tricistronic plasmid. Figure S2: SEC-HPLC analysis of IgGκλ purified from the supernatant of stable CHO pools. Table S1: IgG titers, form distributions and aggregates in the supernatants of stable CHO pools. Table S2: HIC analysis of IgG form distribution.
