# Supplementary material for: Tuning Relative Polypeptide Expression to Optimize Assembly, Yield and Downstream Processing of Bispecific Antibodies
Source: Antibodies (Basel). 2018 Aug 10;7(3):29. doi: 10.3390/antib7030029 (PMC6640677; doi:10.3390/antib7030029)
Supplement: Supplementary file 1 [file antibodies-07-00029-s001.pdf]

# Figure S1

## A

```
                           10        20        30        40        50        60        70        80        90
                            *         *         *         *         *         *         *         *         *
CL wild-type      GGTCAGCCCAAGGCTGCCCCCTCGGTCACTCTGTTCCCGCCCTCCTCTGAGGAGCTTCAAGCCAACAAGGCCACACTGGTGTGTCTCATA
CL docl_1         .....A.....A..C..T..T..T..T...T....T..C..AAGTAGC.....A.....G...........A.....C........G..C
CL docl_2         .....A..G..A..A..A..G..A..A..A..T..T.....G..A..A.....A..C.....A.....A..A..C..C..A..C......
CL docl_3         .....A..G..A..G..A..G.....T..G..A..T.....G..G..G..A..AT.A.....G.....A..G..G..C..C..C......

                          100       110       120       130       140       150       160       170       180
                            *         *         *         *         *         *         *         *         *
CL wild-type      AGTGACTTCTACCCGGGAGCCGTGACAGTGGCTTGGAAAGCAGATAGCAGCCCCGTCAAGGCGGGAGTGGAGACCACCACACCCTCCAAA
CL docl_1         TCC........T..T..G..G..T..C.....C........T......TCT..A..A.....A..C..C.....A.....T.....A..G
CL docl_2         TCA........T.....G..A..A..C..A..A............TCATCA..G.....A..A..G..A..A..T.....C..G..A..G
CL docl_3         TCG..T..T..T.....T..A..A..G..A..G........G...TCGTCG..G.....A.....T..A..A..G..G..G..G..G..G

                          190       200       210       220       230       240       250       260       270
                            *         *         *         *         *         *         *         *         *
CL wild-type      CAAAGCAACAACAAGTACGCGGCCAGCAGCTATCTGAGCCTGACGCCTGAGCAGTGGAAGTCCCACAGAAGCTACAGCTGCCAGGTCACG
CL docl_1         ..GTC.........A.....C..TTCG.........TCTT..........A.........AGT..TC........TCA.....A..G..C
CL docl_2         ..G...........A.....A..A...TCA..C..C.....T..C..G..A..A.....A..A...C.T......TCA.....A..A..C
CL docl_3         ...TCG........A..T.....GTCGTCA..C..ATCG..A.....G..A..A.....A..G..TC.TTCG..TTCG.....A..A...

                          280       290       300       310       320
                            *         *         *         *         *
CL wild-type      CATGAAGGGAGCACCGTGGAGAAGACAGTGGCCCCTACAGAATGTTCATAA
CL docl_1         ..C..G..ATCT..A..C.....A..C.....T..A..T..G.....C...
CL docl_2         ..C......TCA.....A..A..A..T..A..A..G..C..G..CAGC...
CL docl_3         .........TCG..G..A..A..A..G..A..G..G..G........G...
```

## B

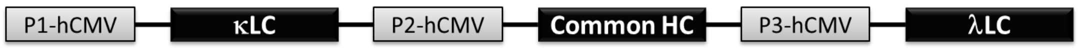

Figure S2

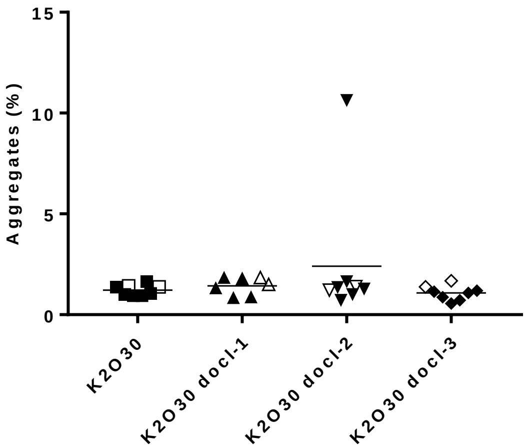

15
10
5
0
Aggregates (%)
K2O30
K2O30 docl-1
K2O30 docl-2
K2O30 docl-3


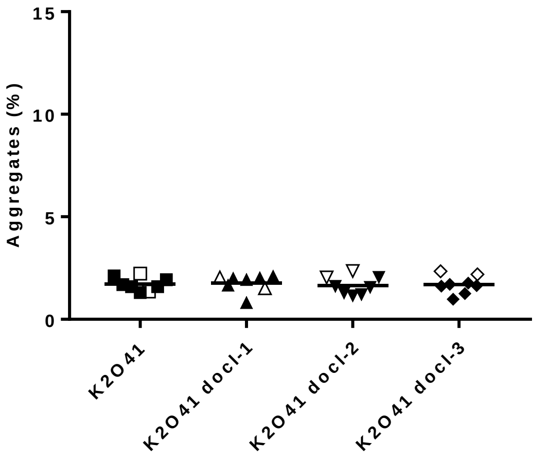

15
10
5
0
Aggregates (%)
K2O41
K2O41 docl-1
K2O41 docl-2
K2O41 docl-3

Table S1. IgG titers, form distributions and aggregates in the supernatants of stable CHO pools.

| | | Total IgG titer (mg/L) | Lambda Light chain (%) | Kappa light chain (%) | IgGκλ (%) | IgGκκ (%) | IgGλλ (%) | IgGκλ titer (mg/L) | Aggregates (%) |
|---|---|---|---|---|---|---|---|---|---|
| K2O41 wt | 1 | 1937.2 | 42.28 | 57.72 | 53.89 | 39.35 | 6.76 | 1043.95 | 2.23 |
| | 2 | 612.4 | 49.26 | 50.74 | 57.01 | 25.72 | 17.27 | 349.12 | 1.59 |
| | 4 | 670.5 | 53.12 | 46.88 | 43.65 | 28.18 | 28.17 | 292.67 | 1.30 |
| | 5 | 783.3 | 43.95 | 56.05 | 54.14 | 32.03 | 13.83 | 424.07 | 1.69 |
| | 6 | 1366.9 | 44.28 | 55.72 | 56.23 | 32.29 | 11.49 | 768.60 | 1.35 |
| | 7 | 810.4 | 43.60 | 56.40 | 54.59 | 28.57 | 16.84 | 442.39 | 1.59 |
| | 8 | 1243.7 | 41.73 | 58.27 | 53.59 | 35.26 | 11.15 | 666.49 | 1.93 |
| K2O41 docl-1 | 1 | 932.9 | 26.01 | 73.99 | 35.41 | 62.55 | 2.04 | 330.34 | 2.11 |
| | 2 | 851.8 | 42.72 | 57.28 | 57.13 | 33.12 | 9.76 | 486.63 | 1.67 |
| | 3 | 2398.4 | 32.16 | 67.84 | 39.14 | 58.61 | 2.24 | 938.73 | 2.04 |
| | 4 | 1161.4 | 31.21 | 68.79 | 37.92 | 58.12 | 3.96 | 440.40 | 1.94 |
| | 5 | 1144.8 | 20.80 | 79.20 | 29.56 | 68.84 | 1.59 | 338.40 | 2.03 |
| | 6 | 2861.8 | 29.44 | 70.56 | 37.61 | 60.68 | 1.71 | 1076.32 | 1.52 |
| | 7 | 1481.0 | 17.42 | 82.58 | 40.87 | 56.06 | 3.07 | 605.28 | 2.01 |
| | 8 | 762.5 | 30.55 | 69.45 | 51.05 | 38.42 | 10.53 | 389.25 | 0.82 |
| K2O41 docl-2 | 1 | 274.5 | 33.16 | 66.84 | 47.70 | 47.89 | 4.41 | 130.93 | 1.15 |
| | 2 | 960.3 | 20.47 | 79.53 | 23.63 | 76.10 | 0.28 | 226.91 | 1.28 |
| | 3 | 1172.1 | 27.07 | 72.93 | 32.64 | 65.91 | 1.44 | 382.57 | 1.55 |
| | 4 | 909.4 | 33.64 | 66.36 | 45.55 | 50.09 | 4.36 | 414.23 | 1.21 |
| | 5 | 1717.8 | 30.13 | 69.87 | 42.53 | 55.51 | 1.96 | 730.58 | 2.35 |
| | 6 | 1525.8 | 34.32 | 65.68 | 45.55 | 49.26 | 5.19 | 695.00 | 2.05 |
| | 8 | 1423.2 | 29.10 | 70.90 | 49.38 | 46.89 | 3.73 | 702.77 | 1.61 |
| K2O41 docl-3 | 1 | 1603.6 | 46.03 | 53.97 | 53.74 | 38.36 | 7.90 | 861.77 | 2.34 |
| | 2 | 675.5 | 49.61 | 50.39 | 57.74 | 30.01 | 12.25 | 390.03 | 1.25 |
| | 3 | 1616.9 | 32.19 | 67.81 | 41.16 | 55.39 | 3.45 | 665.51 | 2.19 |
| | 4 | 1272.8 | 42.42 | 57.58 | 51.33 | 41.59 | 7.08 | 653.32 | 0.98 |
| | 5 | 1432.2 | 54.94 | 45.06 | 57.96 | 29.31 | 12.73 | 830.10 | 1.71 |
| | 6 | 1124.6 | 31.80 | 68.20 | 49.48 | 48.15 | 2.37 | 556.45 | 1.62 |
| K2O30 wt | 2 | 780.1 | 48.80 | 51.20 | 54.88 | 26.65 | 18.47 | 428.11 | 0.94 |
| | 3 | 1668.3 | 30.09 | 69.91 | 31.36 | 65.41 | 3.23 | 523.17 | 1.06 |
| | 4 | 1228.0 | 42.50 | 57.50 | 44.77 | 43.87 | 11.36 | 549.77 | 1.65 |
| | 5 | 926.7 | 44.13 | 55.87 | 53.04 | 33.28 | 13.67 | 491.52 | 1.37 |
| | 6 | 253.9 | 44.70 | 55.30 | 52.45 | 31.29 | 16.26 | 133.17 | 0.95 |
| | 7 | 1075.3 | 54.17 | 45.83 | 61.25 | 22.77 | 15.98 | 658.62 | 1.43 |
| | 8 | 1187.4 | 54.92 | 45.08 | 54.59 | 26.14 | 19.27 | 648.20 | 1.38 |
| K2O30 docl-1 | 1 | 1996.1 | 49.59 | 50.41 | 55.83 | 26.20 | 17.96 | 1114.42 | 1.84 |
| | 3 | 1509.5 | 47.01 | 52.99 | 55.08 | 29.60 | 15.32 | 831.43 | 1.50 |
| | 4 | 1405.3 | 44.95 | 55.05 | 52.71 | 31.99 | 15.30 | 740.73 | 0.84 |
| | 6 | 1163.3 | 44.64 | 55.36 | 48.50 | 33.45 | 18.06 | 564.20 | 1.84 |
| | 7 | 1544.3 | 54.15 | 45.85 | 53.52 | 19.97 | 26.51 | 826.50 | 1.33 |
| | 8 | 366.3 | 47.76 | 52.24 | 54.07 | 25.79 | 20.14 | 198.05 | 0.88 |
| K2O30 docl-2 | 2 | 1530.1 | 33.99 | 66.01 | 43.77 | 49.80 | 6.43 | 669.72 | 1.28 |
| | 3 | 1129.5 | 44.10 | 55.90 | 54.29 | 32.26 | 13.45 | 613.20 | 10.63 |
| | 4 | 1163.1 | 44.38 | 55.62 | 52.64 | 30.05 | 17.31 | 612.25 | 1.65 |
| | 5 | 1743.5 | 38.76 | 61.24 | 51.05 | 38.89 | 10.06 | 890.05 | 1.22 |
| | 6 | 1165.8 | 36.58 | 63.42 | 57.51 | 35.71 | 6.78 | 670.45 | 1.36 |
| | 7 | 1929.1 | 40.20 | 59.80 | 53.20 | 36.96 | 9.85 | 1026.28 | 1.39 |
| K2O30 docl-3 | 1 | 1497.4 | 47.46 | 52.54 | 56.45 | 28.97 | 14.57 | 845.28 | 1.68 |
| | 3 | 610.4 | 36.43 | 63.57 | 48.51 | 43.95 | 7.54 | 296.10 | 0.55 |
| | 4 | 1294.8 | 45.99 | 54.01 | 53.42 | 29.37 | 17.22 | 691.68 | 1.38 |
| | 5 | 614.6 | 35.31 | 64.69 | 56.44 | 37.88 | 5.68 | 346.88 | 0.87 |
| | 6 | 855.3 | 41.64 | 58.36 | 53.78 | 33.21 | 13.00 | 459.98 | 1.20 |
| | 7 | 798.1 | 26.32 | 73.68 | 36.77 | 56.73 | 6.50 | 293.46 | 1.08 |

Table S2. HIC analysis of IgG form distribution after two affinity chromatography steps

| Sample | %IgGκκ | %IgGκλ | %IgGλλ |
|---|---|---|---|
| K2O41 wt-6 | 0,05 | 86,8 | 13,2 |
| K2O41 docl-3-5 | 0,03 | 72,6 | 27,3 |
| K2O41 docl-1-3 | 0,05 | 94,4 | 5,5 |
| K2O41 docl-1-6 | 0,09 | 94 | 6 |
